# Precision Detection and Assessment of Ash Death and Decline Caused by the Emerald Ash Borer Using Drones and Deep Learning

**DOI:** 10.3390/plants12040798

**Published:** 2023-02-10

**Authors:** Sruthi Keerthi Valicharla, Xin Li, Jennifer Greenleaf, Richard Turcotte, Christopher Hayes, Yong-Lak Park

**Affiliations:** 1 Lane Department of Computer Science and Electrical Engineering, West Virginia University, Morgantown, WV 26506, USA; 2 Division of Plant and Soil Sciences, West Virginia University, Morgantown, WV 26506, USA; 3 USDA Forest Service, Forest Health Protection, Morgantown, WV 26505, USA

**Keywords:** average precision, deep learning, drone, emerald ash borer, instance segmentation, invasive species, Mask2former

## Abstract

Emerald ash borer (*Agrilus planipennis*) is an invasive pest that has killed millions of ash trees (*Fraxinus* spp.) in the USA since its first detection in 2002. Although the current methods for trapping emerald ash borers (e.g., sticky traps and trap trees) and visual ground and aerial surveys are generally effective, they are inefficient for precisely locating and assessing the declining and dead ash trees in large or hard-to-access areas. This study was conducted to develop and evaluate a new tool for safe, efficient, and precise detection and assessment of ash decline and death caused by emerald ash borer by using aerial surveys with unmanned aerial systems (a.k.a., drones) and a deep learning model. Aerial surveys with drones were conducted to obtain 6174 aerial images including ash decline in the deciduous forests in West Virginia and Pennsylvania, USA. The ash trees in each image were manually annotated for training and validating deep learning models. The models were evaluated using the object recognition metrics: mean average precisions (*mAP*) and two average precisions (*AP50* and *AP75*). Our comprehensive analyses with instance segmentation models showed that Mask2former was the most effective model for detecting declining and dead ash trees with 0.789, 0.617, and 0.542 for *AP50, AP75,* and *mAP*, respectively, on the validation dataset. A follow-up in-situ field study conducted in nine locations with various levels of ash decline and death demonstrated that deep learning along with aerial survey using drones could be an innovative tool for rapid, safe, and efficient detection and assessment of ash decline and death in large or hard-to-access areas.

## 1. Introduction

As a member of the Oleaceae family, ash trees (*Fraxinus* spp.) are known for their beauty and timber and are economically and ecologically important species [1,2]. Ash trees are distributed primarily in the Northern Hemisphere and some tropical forests in Mexico and Java. Approximately 18 species of ash are found in the USA, of which several are valued for the wood they produce [2]. They comprise approximately 7% of the forests in the eastern USA, and the most commonly used species for timber are white ash (*F. americana*) and green ash (*F. pennsylvanica*) [1,2]. White and green ash trees are scattered throughout the eastern and central parts of the United States, extending towards the southern parts of Canada [2]. The wood of these species is known for its resilience, stiffness, and being lightweight. This wood is used to make a wide range of products, such as baseball bats, pool cues, and electric and acoustic guitars. In addition to these benefits, ash seeds are used to feed various animals and insects. Green ash trees are planted as landscape trees and have thus proven to be a valuable asset for wetlands.

Most ash trees are deciduous, but subtropical ash species are evergreen. The leaves are compound in pinnate form and are divided into 7–15 oval leaflets with a dark green color. The leaves are arranged around the central vein (i.e., opposite composite leaves) and have sharp teeth. They appear around late May and turn yellow in the fall. Ash trees can grow in almost any soil, preferably deep, fertile, and well-drained. Throughout the northeastern USA, there has been a significant increase in ash decline and death. This could be caused by disease or other environmental or abiotic stressors, but one of the greatest threats to ash trees is the emerald ash borer, *Agrilus planipennis* (Coleoptera: Buprestidae). Emerald ash borers are an invasive wood-boring beetle native to the Russian Far East, China, Japan, North Korea, South Korea, Mongolia, and Taiwan [3]. It is thought that emerald ash borers were introduced through the movement of infested wood packaging materials from China and were first detected in the USA near Detroit, Michigan, in 2002 [3]. Currently, emerald ash borers are reported in 35 eastern and mid-western states. Emerald ash borers lay eggs in the cambium, and the larval feeding girdles the tree to death. Once emerald ash borers infest the tree, it takes around 2 years to observe symptoms, such as dead crowns. Eventually, ash tree decline leads to the death of the branch tip and defoliation; the complete death may take place in 2–4 years [4,5,6]. Delaying removal might lead to more fragile branches, which may lead to the falling of the limbs. As removing these declining or dead branches is extremely difficult in natural forests, detection and precise assessment of the ash decline are needed to mitigate and prevent long-term damage. 

During the past 20 years, artificial intelligence including machine learning and deep learning capabilities has been developed at a level that pest management practitioners can use for the surveillance and detection of invasive pests. Image segmentation is the task of converting a digital image into regions of pixels sharing some common characteristics (i.e., segments). Based on the grouping of different semantics, segmentation can be classified as semantic, instance, or panoptic segmentation. Semantic segmentation is per-pixel classification, and the models are based on Fully Convolutional Networks (FCNs) [7]. Instance segmentation is a combination of semantic segmentation and object detection tasks. Object detection architectures, such as You Only Look Once (YOLO) [8], YOLOv2 [9], YOLOv3 [10], You Only Look At CoefficienTs (YOLACT) [11], Single Shot MultiBox Detector (SSD) [12], and RetinaNet [13], localize the objects with bounding boxes, and the semantic segmentation architectures (FCN [7], Deeplab [14], SegNet [15], U-nets [16], Maskformer [17], and Mask2former [18]) provide labels for every pixel. The fusion of object detection and semantic segmentation yields the detection of all instances of a specific category in each image. One of the ground-breaking works for the instance segmentation task is Mask Region-based Convolutional Neural Networks (Mask R-CNN) architecture, which generates masks from the predicted bounding boxes [19]. 

Adapting deep learning models with drone-based remote sensing technologies is emerging as a potential tool for automatic plant health monitoring. During the past years, more sophisticated network architectures were developed based on CNNs and transformers [20] as FCNs led to the loss of information due to their max-pooling layers, which reduced the spatial resolution. Despite its limitations, FCNs, along with SegNets, were adopted for rice crop identification [21,22] and sunflower identification [23] using aerial imageries acquired with drones. Transformer architectures are initially proposed as an improvement over Recurrent Neural Networks (RNNs) and Long Short-Term Memory (LSTM) [24] in the discipline of Natural Language Processing (NLP). These transformer models weigh the significance of each input part differently, and this process is called the attention mechanism. The transformer architecture multiplies the input by a linear transformation to obtain three sets of vectors, namely query vectors (*Q*), key vectors (*K*), and value vectors (*V*). When the attention is calculated on *Q*, *K*, and *V* vectors generated from the same embedding, it is called self-attention, whereas if the attention is calculated based on *Q* of one embedding and *K* and *V* from another embedding, it is termed as cross attention. Inspired by the transformers’ efficiency in NLP tasks, the adaptation of these transformer architectures in the field of computer vision is becoming popular (i.e., ViT [25], DeiT [26], and SWIN [27]). 

Foresters and land managers seek a tool that is efficient and logistically simple not only for the early detection of invasive pests but also for long-term forest health monitoring. Currently, emerald ash borer adults, larvae, pupae, and associated signs and symptoms are targeted in monitoring and surveillance for early detection and rapid response [5,6]. The surveillance generally includes visual checking of ash trees looking for signs of exit holes and leaf feeding, and canopy dieback or death as well as trapping adult emerald ash borers using purple traps with volatile pheromones to attract males or larvae using trap trees [6]. One of the novel tools that can help detection and precise assessment of ash decline and death would be using high-resolution aerial imagery, which can cover a large area or hazardous locations (e.g., cliff) where ground surveys are limited. Several previous studies examined the potential of ash tree identification or ash decline using remotely sensed spectral data acquired from satellites or aircraft. Fraser et al. [28] used hyperspectral sensors and non-parametric algorithms for ash tree identification, and Chan et al. [29] demonstrated that hyperspectral sensors in a manned airplane could distinguish ash dieback caused by a fungus (*Hymenoscyphus fraxineus*). Sapkota and Liang [30] and Murfitt et al. [31] used satellite-based spectral data to map and assess ash decline. Although these previous studies provided excellent insights into using spectral sensors and image analysis for the identification of ash trees and detection of ash declines, a field method is still needed for foresters and pest management practitioners to conduct real-time, in-situ, safe, automated (low logistical need), and high precision surveys. 

Therefore, we developed and tested the capability of deep learning coupled with high-resolution aerial images acquired with drones to detect ash decline and death by determining the efficiency of transformer-based deep learning architecture. We conducted a 2-year field survey to develop a deep learning tool (i.e., CNNs and Transformers using the state-of-the-art deep learning model called Mask2former) to assist land managers and foresters in the precise assessment of ash declines and death. We hypothesized that aerial surveys with drones to acquire aerial images could detect ash decline and death, and the deep learning capability could enhance detection with automation. Specifically, we developed and tested the deep learning models with aerial survey data obtained with drones and validated their capabilities of detecting ash decline and death. In addition, we conducted an in-situ field validation study to further test the deep learning capabilities along with aerial surveys with drones and its applicability to in-situ detection and generation of spatial distribution maps of ash trees. 

## 2. Results

### 2.1. Ash Decline and Death Assessed by Ground Survey 

A total of 6174 images were annotated for generating training (70% of the images), validating (20% of the images), and testing (10% of the images) image data for deep learning (Table 1). 

**Figure 1:**
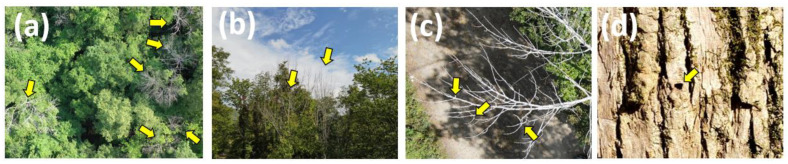
Example aerial view of declining or dead ash trees from aerial images obtained with drones (**a**), ground view of declining or dead ash trees (**b**), and a noticeable opposite branching pattern of ash trees on the aerial images (**c**). The ground survey confirmed the presence of emerald ash borers with adults’ exit holes (**d**). Yellow arrows in (**a**,**b**) indicate declining and dead ash trees, and arrows in (**c**,**d**) indicate an opposite branching pattern and D-shaped exit holes made by adult emerald ash borers, respectively.

Our ground survey confirmed that all the annotated trees for deep learning were indeed declining or dead ash trees. Most of the ash trees (Figure 1a–c) had D-shaped exit holes indicating adult emerald ash borer emergence from the infested trees (Figure 1d), which further indicated the high emerald ash borer infestation causing ash decline and death at the sites.

### 2.2. Evaluation of Instance Segmentation Models in Deep Learning

Using the annotated data (see Figure S1 for example), we trained deep learning models for instance segmentation tasks. To analyze the performance of the instance segmentation models, Microsoft Common Objects in Context (MSCOCO) [32] evaluation metrics including mean average precisions (*mAP*) and the threshold average precisions at 50% and 75% (*AP50* and *AP75*, respectively) were used. In addition, Intersection over Union (IoU), precision, and recall were used to further evaluate the model performance. IoU or the Jaccard index is the ratio of the intersection of the actual and predicted bounding box to their union. IoU gives the correctness of the prediction, precision gives the positive predictive value, and recall gives the true positive rate.

$$IoU=\frac{Area\;of\;overlap}{Area\;of\;union}$$

$$Precision=\frac{True\;Positive}{True\;Positive+False\;Positive}$$

$$Recall=\frac{True\;Positive}{True\;Positive+False\;Negative}$$

The area under the Precision–Recall curve, P–R, gives the Average Precision (*AP*) at IoU threshold *T* (for *AP50*, *T* = 50% and for *AP75*, *T* = 75%), which can be calculated as:

$$AP_{T}=\overset{1}{\underset{0}{\int }}P\left(r\right)dr$$

The mean *AP* (*mAP*) for all the classes is given by:

$$mAP=\frac{1}{All\;classes}\sum AP\left(class\right)$$

Since the number of classes is one in our dataset, *mAP* is calculated for a single class (i.e., ash trees).

### 2.3. Deep Learning for Declining and Dead Ash Tree Detection

We comprehensively examined the performance of the ash tree dataset with various object recognition models. Specifically, we trained the object detection model (i.e., RetinaNet) and instance segmentation models (i.e., Yolact and Mask R-CNN), and then compared their performances with the Masked-attention Mask transformer (Mask2former) model. Mask2former with Swin transformer’s small (SWIN-S) variant as backbone outperforms the other state-of-the-art baselines like Mask R-CNN for all *AP50*, *AP75*, and *mAP* metrics by +0.056, +0.128, and +0.118, respectively, for bounding boxes and +0.16, +0.208, and +0.161, respectively, for segmentation masks (Table 2). Therefore, Mask2former was chosen for detecting declining or dead ash trees on the segmentation maps generated from the aerial images obtained by drones.

### 2.4. Ablation Study of the Mask2former Model

To further analyze the performance of Mask2former, we have employed different CNN and transformer backbones to extract the features from each image. Specifically, we studied the performance of Mask2former on our dataset using ResNets with 50 and 101 layers and Swin transformer’s tiny (SWIN-T), and small (SWIN-S) variants. Furthermore, we used ImageNet-1K [33] weights for all of the backbones.

The results of the test showed that the Mask2former model with ResNet-101 backbone outperforms the SWIN-S transformer model for bounding box *mAP* by +0.017 (Table 3). Whereas for mask *mAP*, SWIN-S was a better choice as it surpassed the ResNet-101 model by +0.005. 

**Figure 2:**
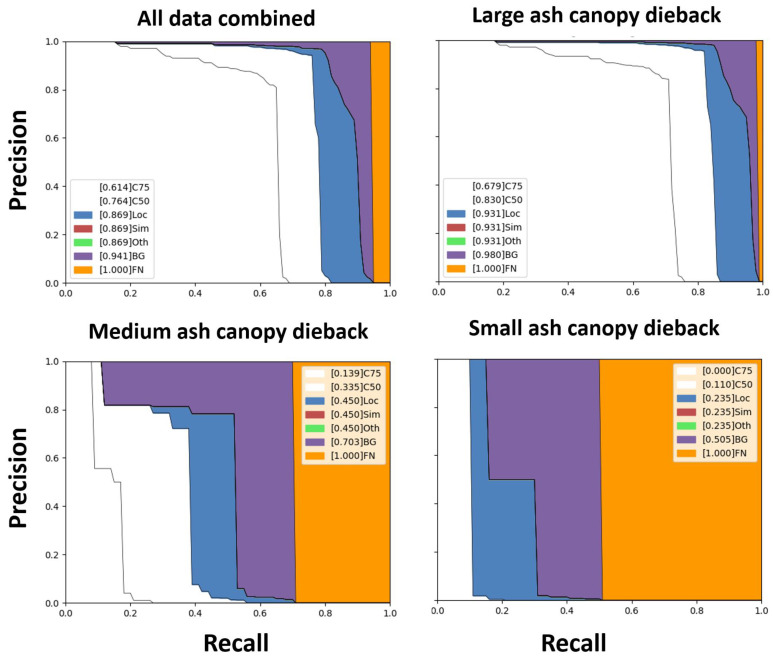
Precision–Recall (P–R) curves for ash trees showing different sizes (large, medium, and small) of ash canopy dieback. C75, IoU = 0.75; C50, IoU = 0.5; Loc, Localization; Sim, Similar objects; Oth, Other; BG, background; FN, false negative. The sizes of the canopy dieback were categorized based on pixel size: small (area < 32^2^ pixels), medium (32^2^ pixels < area < 96^2^ pixels), and large (area > 96^2^ pixels).

In addition to the quantitative analysis, we performed a detailed analysis of the false positives using Precision–Recall (P–R) curves [34]. We classified the declining or dead ash trees into small (area < 32^2^ pixels), medium (32^2^ pixels < area < 96^2^ pixels), and large (area > 96^2^ pixels) objects of the segmentation mask. Each plot comprised a series of P–R curves, including the standard P–R curves at IoU = 0.75 (C75), IoU = 0.5 (C50), and false negative (FN) P–R, which was obtained by removing all the errors making the total area under the curve 1.00. In addition to these P–R curves, based on the differences between the predicted bounding box and the associated ground-truths, the incorrect detection results were given by localization (Loc), similar objects (Sim), others (Oth), and background (BG) P–R curves. Loc is the P–R at IoU = 0.1, ignoring the localization errors. Sim provides the P–R after the super category false positives (FPs) are removed, whereas Oth provides the P–R after eliminating all class confusion. Loc, Sim, and Oth were the same in our case, as we had a single class (i.e., ash tree) instead of multiple classes. Finally, BG gives the P–R when all background FPs are removed. It is evident that C75 and C50 are much higher for larger ash trees than for medium and small trees, indicating that performance was degraded for medium and smaller areas of ash decline and death (Figure 2). 

### 2.5. Visualization of Predictions of Declining or Dead Ash Trees Using Mask2former

**Figure 3:**
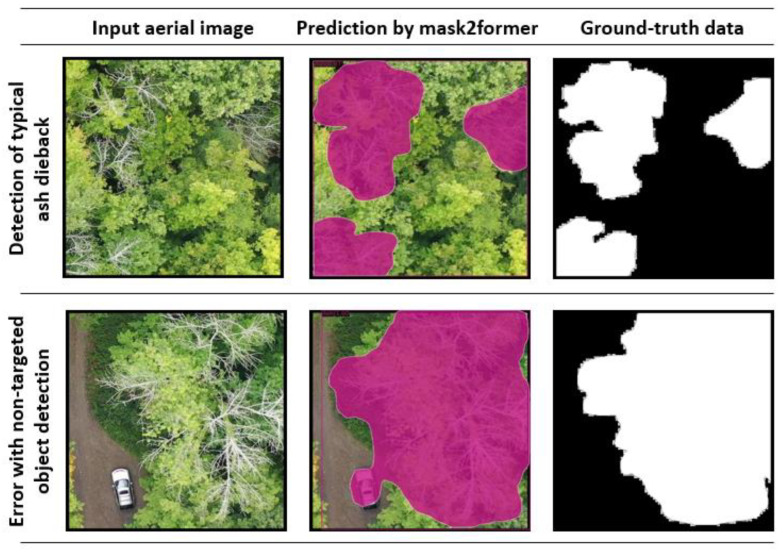
Two examples of Mask2former’s predictions of declining or dead ash trees with a threshold of 0.75. Input aerial images were given to the model, and then the prediction was compared with the ground-truth data.

We tested our model’s performance by conducting a validation with the testing dataset. For the testing dataset, we used 0.75 as a threshold to detect the presence of the declining or dead ash tree. The prediction results of the testing dataset showed that the model performed well in detecting declining or dead ash trees (Figure 3). However, cars or human-made objects were sometimes predicted to be declining or dead ash.

### 2.6. In-Situ Field Validation Study 

**Figure 4:**
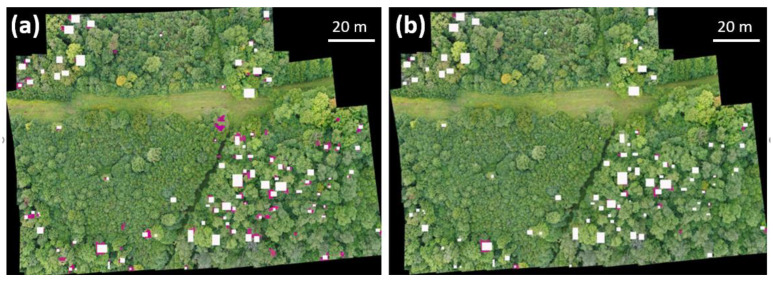
Example in-situ validation for the detection of declining or dead ash trees using Mask2former, a supervised deep learning model with two different confidence levels: 0.1 (**a**) and 0.5 (**b**). White boxes indicate declining or dead ash trees located by ground survey, and purple areas (bounding box) indicate declining or dead ash trees detected and located by Mask2former. By comparing ground survey data with Mask2former prediction map, type I and II errors could be calculated.

To demonstrate the ability of Mask2former along with the use of drones for aerial surveys to detect declining or dead ash trees in a variety of environmental conditions, we conducted aerial surveys at nine sites with drones for in-situ field validation. We used lower thresholds (i.e., 0.1 and 0.5) as the field validation images are dissimilar in terms of lighting and altitudes when compared to the training data used for training the deep learning model. A composite image of each site was subjected to automated detection of declining or dead ash trees (Figure 4). The results of this study showed that the Mask2former model correctly detected 81% ± 10.7 of declining or dead ash trees ranging from 59% to 100% at the confidence level of 0.1; the model did not detect 19% of declining or dead ash trees (i.e., false negative or type II error). A total of 30% ± 33.1 trees (ranging from 0% to 83%) were falsely detected as declining or dead ash trees (i.e., false positive or type I error) at the confidence level of 0.1. At the confidence level of 0.5, the model detected correctly 27% ± 13.6 of declining or dead ash trees but detected falsely 6% ± 8.8 (i.e., type I error). We also noticed no false positive detection of declining or dead ash trees in four of the nine sites (see Table S1 for detailed data).

## 3. Discussion and Conclusions

This study aimed at developing an application with deep learning and automated drone operation to precisely assess the ash decline and death caused by emerald ash borers. Specifically, this study demonstrated that a deep learning model (i.e., Mask2former) coupled with aerial images acquired with autopiloted drones could generate distribution maps of ash decline and death, providing detailed images and geo-coordinates of individual ash trees for foresters and land managers to use for their management planning and action. In this study, we first tested various deep learning models and found that Mask2former, a supervised deep learning model, performed the best for the instance segmentation task on the ash tree images. Most previous studies on crop detection using aerial images focused on classification [28] or semantic segmentation tasks [22,23]. However, classification models cannot accurately locate the ash trees, and the semantic segmentation models mark all the ash trees as one single ash tree. Therefore, our model was trained to perform instance segmentation tasks by marking each ash tree as a separate instance. Moreover, we used a transformer decoder with a masked attention mechanism to extract the localized features from the ash tree images by constraining the cross attention within the foreground regions of the predicted mask. Based on our results, ash trees with more canopy dieback were detected more precisely and the performance of the model was degraded for medium and smaller symptoms of ash decline and death. 

**Figure 5:**
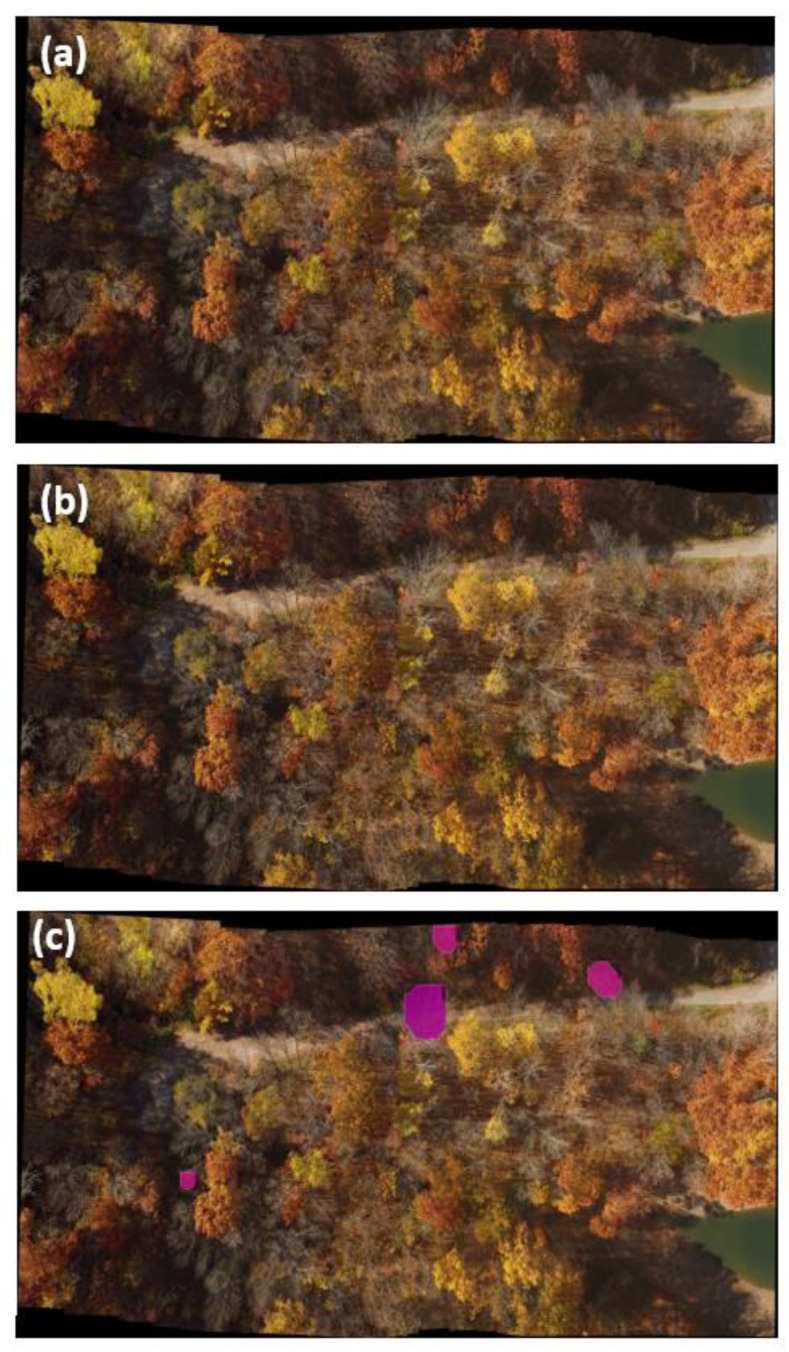
Prediction of Mask2former model on images taken in autumn; (**a**) is an input image, and (**b**,**c**) are the predictions with a threshold of 0.5 and 0.1, respectively.

Although our deep learning model worked well with unseen testing data, the model failed to predict ash decline in the images with seasonal change (e.g., autumn season). For example, we tried to predict the dead or declining ash trees by feeding the autumn season images to the deep learning model (Figure 5a). With a threshold of 0.5 (Figure 5b), the model could not detect any of the dead or declining ash trees, but it detected only a few of the declining ash trees with a threshold of 0.1 (Figure 5c). We hypothesize that the cause for this deficiency is imprecise manual annotations around the possible ash trees, which include some green regions. As a result, the model failed to detect ash tree in autumn as the leaves are no longer green. Furthermore, the Mask2former failed to detect smaller ash trees on the aerial images taken.

The results of our aerial surveys with drones were very promising. The aerial images were high-resolution (4K), and even individual branches (declining or dead) of an ash tree could be examined (see Figure S3 for example). Our study suggests that pixel-based modeling would be possible because C75 and C50 were much higher for larger ash trees than for medium and small trees, indicating that performance was degraded for medium and smaller areas of ash decline and death (Figure 2). In addition, the in-situ validation study also demonstrated a promising ability to detect declining or dead ash trees. We trained our model on images captured 50–70 m above the ground in summer and tested its performance in nine sites in Pennsylvania and West Virginia, USA. We used lower thresholds (0.1 and 0.5) for in-situ field validation due to the dissimilarity in drone-flight altitudes and lighting compared to the training images. We found a higher type I error (29%) and lower type II error (19%) at the threshold of 0.1. Type I error (i.e., false positive) is generally acceptable in pest management because it indicates that no ash trees are declining or dead even though the model’s output informs foresters or land managers to visit and check falsely detected declining or dead ash trees. However, type II error (false negative) is more critical for pest management because there are declining or dead ash trees, but the model’s output indicates there are no declining or dead ash trees. In this case, foresters and land managers may not check the false-negative trees which can be a source of future spread of ash decline and death by emerald ash borer. At the confidence level of 0.5, the type II error was higher (71%), which may not be acceptable for pest management although the type I error was only 6%. In addition, we noticed that type I and II error rates varied by site (see Table S1 for details). Four of the nine sites had 0% type I error while the other sites had as high as 83% type I error at the precision of 0.1; only one site had 0% type II error. Therefore, to develop a tool that is applicable to a wide variety of forests, deep learning models need training data acquired from various environments and locations. 

Although our study is the first to use high-resolution drone imagery and the Mask2former deep learning model together, previous studies used satellites and spectral sensors to identify ash trees and detect ash dieback. Spectral analyses from the previous studies [29,30,31,35] showed promising results for the detection of ash trees infested with emerald ash borers although the studies also found some limitations in using spectral sensors and satellites. Four main limitations were: (1) the differences in spectral reflectance between the leaves of infested and non-infested ash trees are sometimes difficult to distinguish, (2) emerald ash borer data were collected one time during the growing season while the factors that affect spectral response can vary across a growing season, (3) the high cost of the hyperspectral data may prohibit use by many foresters and land managers, and (4) some ash trees were confused with other trees because the ash detection is based on the classification maps. In addition to satellites, piloted or manned airplanes have been used for aerial visual surveys (e.g., aerial sketch mapping), which can cover a large area but still provide acceptable precision for forest health monitoring [28,36,37,38]. Aerial sketch mapping methods are well adopted by federal and state agencies in the USA to cover large areas in a short period [39,40]. However, aerial sketch mapping methods are restricted by weather, cost, availability of airplanes and pilots, and safety. 

Drones have become an excellent platform for easy, cheap, high-resolution, and real-time aerial surveys [41]. With the continuing advancement and growing application of drones, foresters and land managers have used them for evaluating and monitoring forests and even individual trees [28]. The application developed from this study using deep learning and drones generates distribution maps of declining and dead ash trees. The maps can help locate areas with declining and dead ash trees, especially in very dense large forests or hard-to-access areas. Even, the method we developed in this study can identify the location (i.e., geo-coordinates) of individual ash trees, which can help foresters and land managers develop an efficient forest health monitoring plan or survey route for periodical or repeated surveys of declining ash trees by using pre-planned, autopiloted drone flights [39,41]. In addition to mapping ash declines, drones can be used with ground robots or surveyors to enhance the real-time detection of ash decline and death. Using aerial surveys with drones (even with satellites and piloted airplanes), it is extremely difficult to identify the cause of ash decline and death based on the symptoms shown on the top of the canopy. The symptoms are visible from the sky when the upper branches of ash trees are deprived of water and nutrients, and thus the trees are defoliated, and parts of the crown lose leaves in spring and summer [35]. However, the symptoms of emerald ash borer damage can be similar to those of other environmental factors and cause agents such as fungal pathogens [35]. The presence of emerald ash borers is evidenced by the D-shaped exit holes in the rough and grooved ash bark and the larval tunneling under the bark [1], indicating a need for a ground survey. In addition, the visual symptoms of emerald ash borer infestation do not usually appear until 2–4 years after the arrival of emerald ash borers [40], and thus foresters and land managers may not notice signs of infestation until they begin to see bare branches [1]. Our study using aerial surveys did not attempt to detect emerald ash borer directly, but the methods developed in this study can guide foresters and land managers on where to go to find declining or dead ash trees with the ground survey or low-altitude drone flights to confirm and assess the ash decline and death. The methods also would help prioritize areas for the management of ash decline and emerald ash borer.

Our study was not designed to detect the early stage of ash decline by showing symptoms of yellowing or direct detection of insects; previous studies with hyperspectral sensors showed the potential for early detection of ash decline [28,29,30], which is encouraging the use of drones and hyperspectral sensors together to enable early detection and rapid response to emerald ash borers. Our study instead focused on the precise detection of ash decline and death using deep learning and drones together to develop a method that is (1) low logistics, (2) automated, (3) cheap, (4) safe, (5) using real-time methods, and (6) covering hard-to-access areas. These advantages overcome the limitations of satellite- and airplane-based forest health monitoring. A major disadvantage of the use of drones would be the limited coverage of survey areas compared to satellites and airplanes. However, the method we developed in this study can be combined with satellites or piloted airplanes, which would enhance the detectability and overcome the pros and cons of each method (i.e., data fusion [28]). Data or image fusion allows users to overcome the shortcomings of single data source limitations. For example, with the fusion of satellite and drone imagery, foresters and land managers could overcome the low spatial resolution of most satellite sensors and the limited coverage that can be accomplished by drones [28,41]. In addition, deep learning in artificial intelligence, which emerged as one of the potential tools for visual recognition tasks, could add to the data fusion and be used for short-term and long-term forest health monitoring. Federal and state agencies in the USA are currently using drones for routine forest health monitoring. As more aerial image data will be collected as a routine task, the data can be added to our deep learning model for training and validation, which eventually increases the precision and accuracy of the deep learning model. In addition, this method can be applied to detect trees damaged by other invasive pests such as insects, diseases, or weeds.

Although this study demonstrated the potential for the use of drones and deep learning together to overcome current limitations in the assessment and management of ash decline and death, further studies are needed to increase the precision and accuracy of the detection of ash decline and death caused by emerald ash borders. Because other environmental or biotic stressors (e.g., *H. fraxineus*, a fungal pathogen in Europe) also can cause ash decline and death, additional studies to differentiate the causal agents would be necessary. In addition, we aim to improve the process of marking the ash trees by incorporating multi-spectral, hyperspectral, and thermal data (i.e., fusion techniques) into our dataset along with the usage of dilated backbones to segment smaller objects, thereby making our model robust. Furthermore, we plan to expand the database of our ash tree dataset by collecting the images with seasonal change and re-train our model using transfer learning.

## 4. Materials and Methods

### 4.1. Study Sites and Types of Aerial Surveys

**Figure 6:**
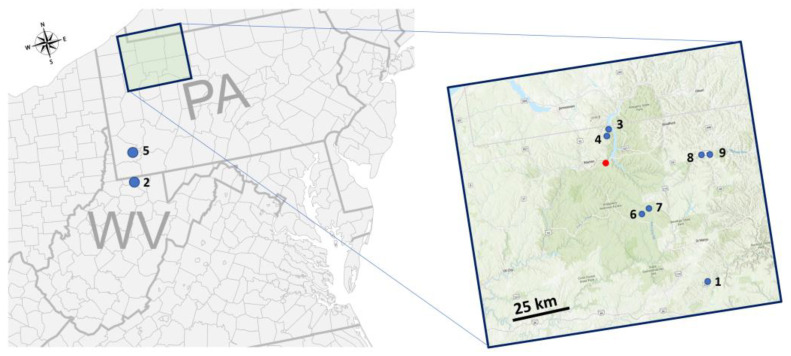
Locations of study sites (blue and red dots) in Pennsylvania and West Virginia, USA. The red dot indicates the site where data for deep learning were collected, and the blue dots indicate in-situ validation study sites.

This study was conducted in Pennsylvania and Northern West Virginia, USA (Figure 6). The size of the sites raged from 1.8 ha to 36 ha. Ash trees in these study sites had experienced emerald ash borer infestations for at least 5 years, causing severe decline and death of ash trees. Two separate sets of aerial surveys were conducted in this study: aerial surveys for obtaining data for deep learning (see Section 4.2 and Section 4.3) and in-situ validation surveys (see Section 4.6). 

### 4.2. Acquisition of Aerial Imagery Using Drones for Deep Learning

Aerial surveys for deep learning were conducted in a 20-ha area located in Warren County, Pennsylvania, USA, during the summers of 2020 and 2021. Various levels of ash tree decline and death have been found in the area, making it an ideal area to obtain aerial images for deep learning. 

Two rotary-wing drones (DJI Mavic 2 Enterprise Advanced and DJI Mavic 2 Pro; SZ DJI Technology Co., Ltd., Shenzhen, China) were used. The DJI Mavic 2 Enterprise Advanced carried a 3.3-megapixel thermal camera as well as an RGB camera with 48-megapixel photography and 4K videography capability. The DJI Mavic 2 Pro carried an RGB camera with 20-megapixel photography and 4K videography capability.

The drones were flown 50–75 m above the ground depending on the terrain and obstacles in the study sites. Our preliminary study showed that aerial surveys with 4K videography at <80 m above ground could detect declining or dead ash trees (Figure S3). After the flight missions were completed, aerial images were downloaded from the drone and aligned according to the pre-planned flight path.

### 4.3. Data Processing and Labeling

**Figure 7:**
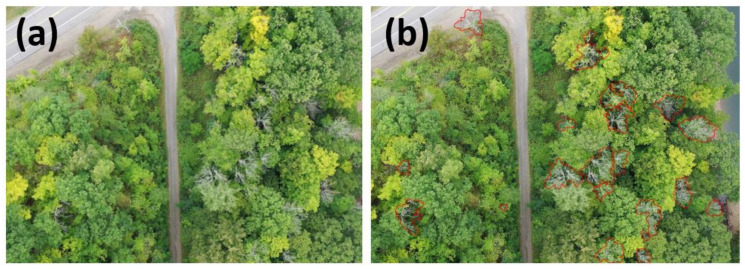
An example of the original RGB image (**a**) and its manual annotations (**b**) of declining and dead ash trees, which are outlined in red.

From the 4K video footages from the drones, 3423 images were captured. Every 10^th^ image frame was chosen for this study to maintain positional variation, and all 343 images were manually annotated using the BeFunky Photo Editor (BeFunky, Portland, OR, USA). Annotating an image with the dead ash trees (Figure 7) took approximately 2 min per image. Ideally, the outline would be tighter to the branches, but that would increase the time required to annotate. Dead or declining ash trees could be distinguished from an aerial view via their oppositely arranged, stout, and relatively straight branches, which can be seen in the ground view image (Figure 7a). They are also particularly white in color. In addition, ground surveys were conducted to confirm that the declining or dead trees were indeed ash trees. 

**Figure 8:**
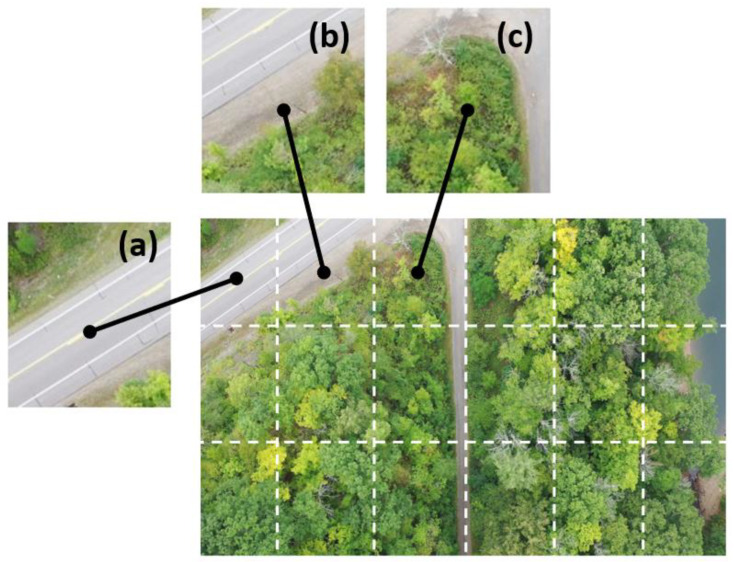
An illustration of patches generated from a 4K image acquired with drones. A total of 18 patches with dimensions of 640 pixels by 720 pixels were extracted from each 4K image for deep learning. Image patches (**a**,**b**) without declining ash trees, and image patch (**c**) has a declining or dead ash tree.

As each image’s dimensions are 3840 pixels × 2160 pixels, it is computationally expensive to train a deep learning model on these 4K images. Therefore, non-overlapping patches with dimensions 640 pixels × 720 pixels were cropped from each 4K image producing 18 patches from each image (Figure 8). This patch extraction leads to many negative samples (i.e., no ash trees) (Figure 8a,b). This yields 6174 images in total for deep learning.

**Figure 9:**
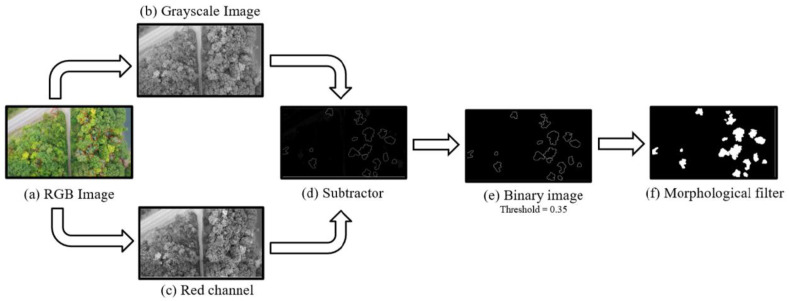
Workflow for binary segmentation mask generation. An RGB image (**a**) is converted into grayscale information of the RGB image (**b**) and red channel information of the RGB image (**c**). Then, a subtracted image (**d**) is used to convert to a binary image (**e**), and the final mask is generated after applying the morphological filter (**f**).

The annotated aerial images were converted into binary masks, which served as the ground-truth for training the deep learning models (Figure 9). These binary masks for ash trees were generated using MATLAB R2021a (The MathWorks Inc., Natick, MA, USA). The workflow (Figure 9) included extracting the red channel and grayscale information of the RGB images (Figure 9b,c), subtracting the grayscale image from the red channel image (Figure 9d), converting the subtracted image to a binary image with a 0.35 threshold that could include the small areas of annotations (Figure 9e), and using a morphological operation to fill up the marked zones after having the outline of the annotations. The final ground-truth mask with the white regions constituted the presence of declining or dead ash trees, and the black areas were the backgrounds (Figure 9f).

**Figure 10:**
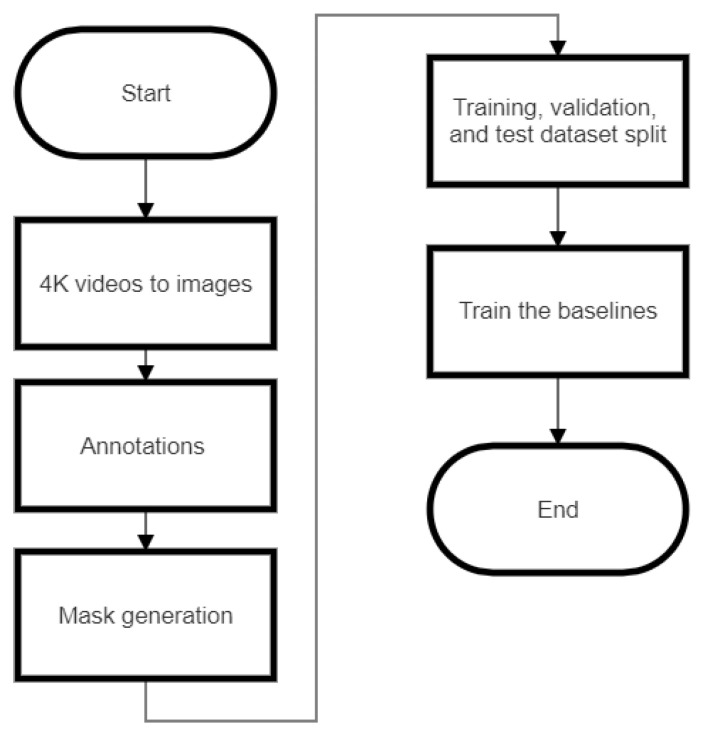
The overall workflow for the prediction of ash tree decline and death.

The dataset for deep learning consisting of 6174 images was split into 4357 training images, 1200 validation images, and 617 test images (7:2:1). As mentioned earlier, each 4K image was divided into 18 patches to make the dataset computationally efficient. Potentially, we could downsize the 4K image, but the downsizing would lead to information loss while generating the annotation masks (Figure S1). Thus, we used image patches with different augmentation techniques. These training, validation, and testing datasets had positive (presence of ash decline and death) and negative (absence of ash decline and death) images. These images and masks were used to create annotations in the standard MSCOCO dataset format. The procedure for the prediction of ash trees from a given video is given as a flowchart shown in Figure 10.

### 4.4. Overview of Deep Learning Procedures

**Figure 11:**
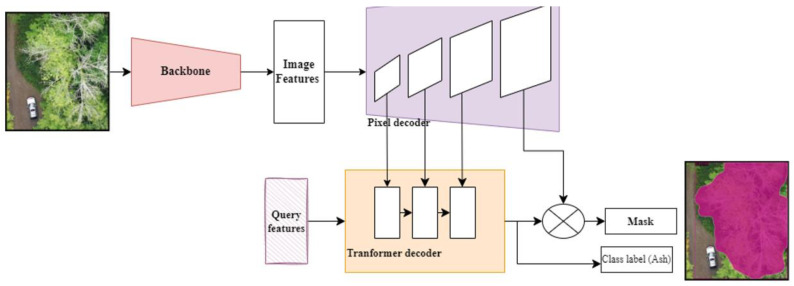
The overall network architecture of the deep learning model (Mask2former). The input RGB image is fed to the backbone to extract low-resolution features. The pixel decoder block upsamples the low-resolution features. The decoder block takes in the image features and queries and provides the segmentation mask and class label. The final output of the model is an RGB image with a bounding box, class label, and a segmentation mask on the predicted dead or declining ash trees.

To detect declining or dead ash trees, we used Mask2former [18]. Unlike Mask R-CNN, the semantic segmentation task is not limited to detected bounding boxes. Instead, each segment in a digital image is converted into a C-dimensional vector (DETR [42]), mask former [17]), and fed to a trained decoder. The architecture consists of a backbone, a pixel decoder, and a transformer decoder with masked attention. The purpose of the backbone is to extract low-resolution features. The pixel decoder upsamples these low-resolution features and converts them into high-resolution per-pixel embeddings. The transformer decoder takes in the image features and converts them into object queries. From these per-pixel embeddings with object queries, the final binary mask predictions are obtained. The input RGB image of the size *W* × *H* was given as input to the backbone to extract the feature map F $\epsilon \;{\mathbb{R}}^{Cx\frac{H}{S}X\frac{W}{S}}$ where *C* was the number of channels, *H* and *W* were the height and width of the input image, and *S* was the feature map’s stride. In this study, we used RGB images so the number of channels would be three, but the stride of the feature map varied with different backbones. We explored CNN-based architectures (ResNet50 and ResNet101 [43]) and transformer-based architecture (SWIN [27]) as backbones for this study. These low-resolution features were then fed to a pixel decoder to gradually upsample and produce per-pixel embeddings ${ℰ}_{pixel}\epsilon \;{\mathbb{R}}^{C_{g}xHxW}$ where *C_g_* was the dimension of the embeddings. The overall architecture is shown in the Figure 11. 

The chief component of the transformer block was the masked attention operator. The function of this operator was to extract features from the predicted mask by limiting the cross attention [20] to just foregrounds instead of the entire feature map. Along with that, the pixel decoder’s successive feature maps were fed to successive transformer decoder layers thereby utilizing the high-resolution features to extract smaller objects. The standard cross attention at any layer l was given as:

*X_l_* = softmax(*Q_l_K_l_^T^*)*V_l_* + *X_l_*_−1_

where *X_l_* refers to the query features at *l*th layer. *Q_l_*, *K_l_*, and *V_l_* are the image features under the linear transformations (i.e., f_Q_, f_K_, and f_V,_ respectively). *X_o_* denotes the transformer decoder’s input query features. By adding the attention matrix to the standard cross-attention, we had:

*X_l_* = softmax(*M_l_*_−1_ + *Q_l_K_l_^T^*)*V_l_* + *X_l_*_−1_

The matrix *M_l_*_−1_ at any feature point (*x*,*y*) gives the binarized mask predicted from the previous transformer layer (*l* − 1)th with a threshold of 0.5.

(1) $$M_{l-1}\left(x,y\right)=\{\begin{array}{cc} 0, & if\;M_{l-1}=1\\ -\infty , & else \end{array}$$

The query features of the architecture of the decoder block (Figure S2) are processed in the following order: cross-attention, self-attention, and feed-forward network (FFN). The query features are affiliated with learnable positional embeddings and are initiated to zero before feeding into the decoder.

### 4.5. Data Augmentation and Training Procedure

Mask2former is a supervised deep learning model. Training such a model requires many image examples with proper annotations to learn complex patterns for higher-level data abstraction. Annotating large datasets is very time-consuming (e.g., 2 min per image in our study). To improve the data efficiency of the deep learning models, we employed data augmentation techniques [44]. We used the large-scale jittering technique (LSJ) [45,46] as our primary data augmentation strategy. We used a fixed crop size of 1024 pixels × 1024 pixels and an image resize technique with a ratio range of 0.1 to 2.0. Along with LSJ, padding and normalization techniques were also used for training the model. We used test-time augmentation (TTA) with multiple scales and flipping provided by mmdetection [47]. Inspired by the standard Mask R-CNN inference settings, the image scale was set to 800 pixels and 1333 pixels on the shorter and longer side, respectively.

We performed all our experiments on a single NVIDIA GeForce RTX 3090. Our implementations are based on mmdetection [47]. Furthermore, we used the AdamW optimizer with a learning rate of 0.0001 and a weight decay of 0.05 for all the baselines. The batch size was set to 2 and the other environment details are as follows: Python 3.10.4, GCC 9.3, CUDA 11.6, PyTorch 1.12.1, MMdetection 2.25.1, CuDNN 8.5, and Ubuntu 20.04.1.

We employed weighted loss functions for calculating the loss for training the model. For mask loss, *L_mask_*, we used binary cross entropy loss with a weight *λ_mask_* = 5.0 and for class loss *L_cls_*, we used the binary cross entropy loss instead of cross entropy (i.e., single class dataset) with weight *λ_cls_* = 2.0. Along with these loss functions, we used Dice loss [48] *L_Dice_* with a weight *λ_dice_* = 5.0. The total loss (L) was a combination of *L_mask_, L_cls_,* and *L_Dice_* and expressed as,

*L* = *λ_mask_* × *L_mask_* + *λ_cls_* × *L_cls_* + *λ_dice_* × *L_Dice_* (2)

### 4.6. In-Situ Field Validation Study

To assess the detection ability of the finalized deep learning models, we conducted an in-situ field validation study. This type of study also implies the applicability of the developed deep learning models to real-world problem-solving. 

A total of nine sites were selected for in-situ validation study, and the sites were located in Pennsylvania (Greene, Warren, Elk, McKean, and Forest counties) and Northern West Virginia (Monongalia County). These sites experienced low to heavy emerald ash borer infestations, leading to the death and decline of ash trees. A detailed description of the sites can be found in Table S1.

**Figure 12:**
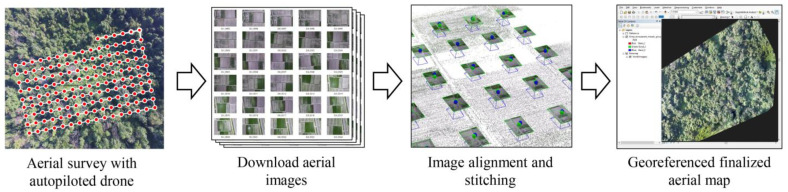
Drone operation and image analysis protocol used in this study.

A protocol to obtain aerial images for the detection of declining or dead ash trees was developed to efficiently and promptly complete the survey. The main steps in the protocol included: (1) a target area for the aerial survey was defined and a drone flight path was planned, (2) a drone was deployed with an autopilot function for surveys with aerial photography over the target area, and (3) aerial images were downloaded and stitched to generate a composite image of the target area (Figure 12). 

The drone was flown 50–75 m above the ground depending on terrain and obstacles. After the flight missions were completed, aerial images were downloaded from the drone and aligned according to the pre-planned flight path. The aerial images were stitched with Pix4DMapper (Pix4D, Prilly, Switzerland) to generate a composite image, which was then georeferenced to confer spatial attributes to the map coordinate system, i.e., ortho-mosaicking with ArcInfo^®^ 10 (ESRI, Redland, CA, USA). 

The composite images were cropped into patches of 640-pixel-by-720-pixel dimensions and were fed to the trained Mask2former model. The output of the model was the series of predictions on the cropped patches. Finally, these predicted patches were stitched together to form the composite image. The final outputs were maps with boundary boxes of declining or dead ash trees.

To compare the outputs of the deep learning model with those of ground-truth data, we conducted ground surveys. The locations of individual declining and dead ash trees were recorded with GPS at each of nine sites. The results of the ground survey were used to validate the deep learning outputs by calculating and comparing the type I error (the aerial survey detected declining or dead ash trees, but these trees were not detected by ground validation) and the type II error (the aerial survey did not detect declining or dead ash trees, but trees were detected by ground survey) [49]. 

## Figures and Tables

**Table 1:** Data used for developing deep learning models including training, validation, and testing datasets.

Data Category	Images with Declining or Dead Ash Tree	Images without Declining or Dead Ash Tree	Total Number of Images
Training	2718 (62.4%)	1639 (37.6%)	4357
Validation	800 (66.7%)	400 (33.3%)	1200
Testing	475 (77.0%)	142 (23.0%)	617

**Table 2:** Assessment of different state-of-the-art baselines and backbones for the detectability of declining or dead ash trees by using mean average precisions (*mAP*) and the threshold average precisions at 50% and 75% (*AP50* and *AP75*, respectively).

Baseline	Backbone	Pre-Train	Bounding Box			Mask		
			*AP50*	*AP75*	mAP	*AP50*	*AP75*	*mAP*
RetinaNet	ResNet-18	ImageNet-1K	0.6630	0.4850	0.3810	-	-	-
Yolact	ResNet-101	ImageNet-1K	0.6640	0.4360	0.4141	0.6270	0.4040	0.3640
Mask R-CNN	SWIN—T	ImageNet-1K	0.6730	0.4640	0.4440	0.6290	0.4090	0.3810
Mask2former	SWIN—S	ImageNet-1K	0.7290	0.5920	0.5620	0.7890	0.6170	0.5420

**Table 3:** Instance segmentation results on the ash tree dataset using Mask2former with different backbones by using mean average precisions (*mAP*) and the threshold average precisions at 50% and 75% (*AP50* and *AP75*, respectively).

Method	Backbone	Pre-Train	Bounding Box			Mask		
			*AP50*	*AP75*	*mAP*	*AP50*	*AP75*	*mAP*
CNNs	ResNet-50	ImageNet-1K	0.7260	0.5630	0.5340	0.7800	0.5930	0.5240
CNNs	ResNet-101	ImageNet-1K	0.7510	0.6150	0.5790	0.7680	0.6170	0.5370
Transformers	SWIN—T	ImageNet-1K	0.7120	0.6220	0.5780	0.7590	0.5720	0.5180
Transformers	SWIN—S	ImageNet-1K	0.7290	0.5920	0.5620	0.7890	0.6170	0.5420

## Data Availability

The data that support the findings of this study will be available upon request.

## Supplementary Materials

The following supporting information can be downloaded at: https://www.mdpi.com/article/10.3390/plants12040798/s1, Table S1: The number of declining or dead ash trees detected by ground survey and the Mask2former (deep learning model), and associated statistics including types I and II errors. This in-situ validation study was conducted in nine different sites in Pennsylvania and West Virginia, USA, and two different thresholds (0.1 and 0.5) for the Mask2former were used; Figure S1. Disadvantages of image resizing in segmentation. (a), RGB image; (b), mask generated after cropping the RGB image into patches; (c), mask generated by resizing the RGB image; Figure S2. Transformer decoder architecture of the Mask2former model. The Queries (*Q*), Keys (*K*), and Values (*V*) vectors derived from the RGB image are fed to the cross-attention block, self-attention block, and the feed-forward network (FFN) to obtain segmentation maps and bounding boxes with a class label; Figure S3. Resolutions of aerial images showing ash tree dieback at different drone flight altitudes. Even 80 m above the ground, aerial images show the opposite branching pattern of declining ash trees.
