# Supplementary material for: Precision Detection and Assessment of Ash Death and Decline Caused by the Emerald Ash Borer Using Drones and Deep Learning
Source: Plants (Basel). 2023 Feb 10;12(4):798. doi: 10.3390/plants12040798 (PMC9964414; doi:10.3390/plants12040798)
Supplement: Supplementary file 1 [file plants-12-00798-s001.zip › plants-2102567-supplementary.pdf]

Table S1. The number of declining or dead ash trees detected by ground survey and the Mask2former (deep learning model), and associated statistics including types I and II errors. This in-situ validation study was conducted in 9 different sites in Pennsylvania and West Virginia, USA, and two different thresholds (0.1 and 0.5) for the Mask2former were used.

Threshold	Site	No. of ash trees detected by ground survey	No. of ash trees detected by Mask2former	% overestimation (+) or underestimation (-) by Mask2former	True positive	False negative (Type II error)	False Positive (Type I error)
0.1	1	18	18	0.0%	77.8%	22.2%	0.0%
	2	22	22	0.0%	59.1%	40.9%	0.0%
	3	26	26	0.0%	84.6%	15.4%	0.0%
	4	40	66	65.0%	100.0%	0.0%	65.0%
	5	76	76	0.0%	86.8%	13.2%	0.0%
	6	52	84	61.5%	80.8%	19.2%	61.5%
	7	93	110	18.3%	80.6%	19.4%	18.3%
	8	100	138	38.0%	77.0%	23.0%	38.0%
	9	63	115	82.5%	82.5%	17.5%	82.5%
0.5	1	18	18	0.0%	16.7%	83.3%	0.0%
	2	22	22	0.0%	36.4%	63.6%	0.0%
	3	26	26	0.0%	26.9%	73.1%	0.0%
	4	40	50	25.0%	37.5%	62.5%	25.0%
	5	76	66	-13.2%	55.3%	31.6%	0.0%
	6	52	60	15.4%	17.3%	82.7%	15.4%
	7	93	94	1.1%	20.4%	79.6%	1.1%
	8	100	105	5.0%	14.0%	86.0%	5.0%
	9	63	68	7.9%	19.0%	81.0%	7.9%

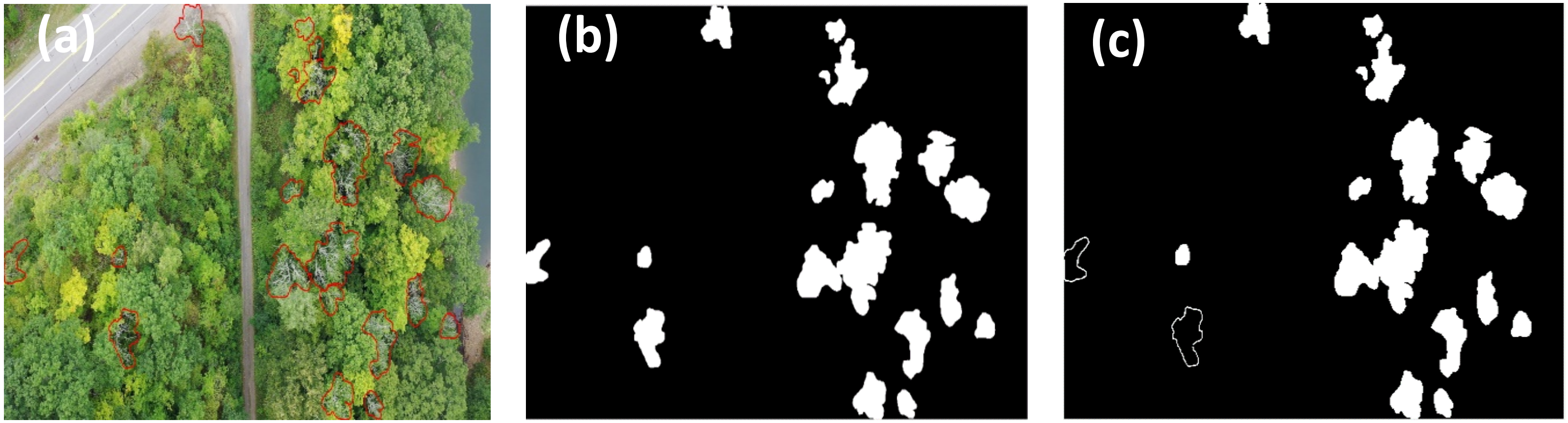

Figure S1. Disadvantages of image resizing in segmentation. (a), RGB image; (b), mask generated after cropping the RGB image into patches; (c), mask generated by resizing the RGB image.

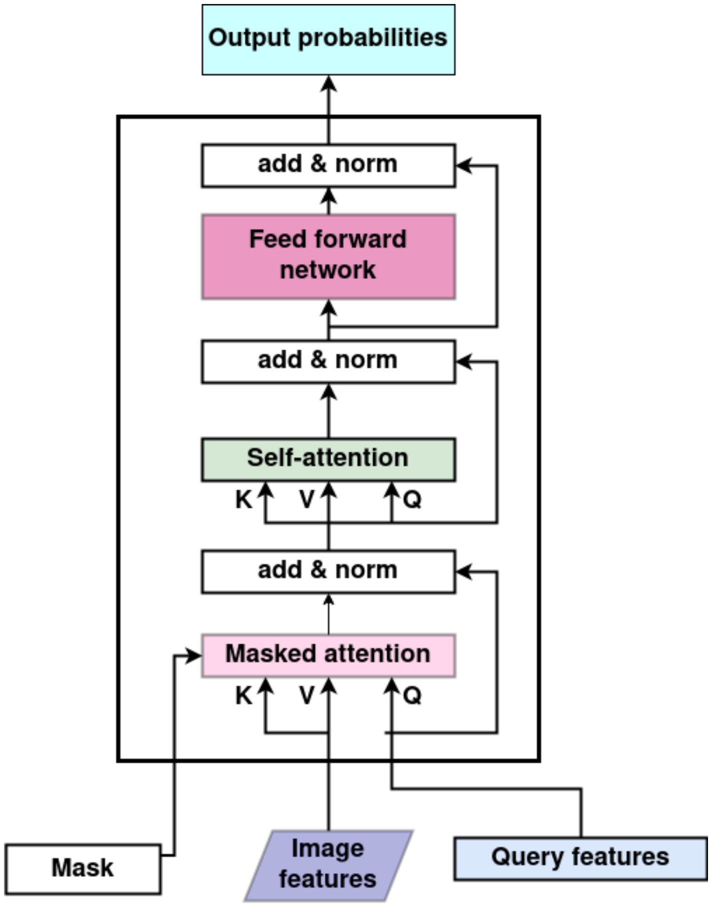

Figure S2. Transformer decoder architecture of the Mask2former model. The Queries (Q), Keys (K), and Values (V) vectors derived from the RGB image are fed to the cross-attention block, self-attention block, and the feed-forward network (FFN) to obtain segmentation maps and bounding boxes with a class label.

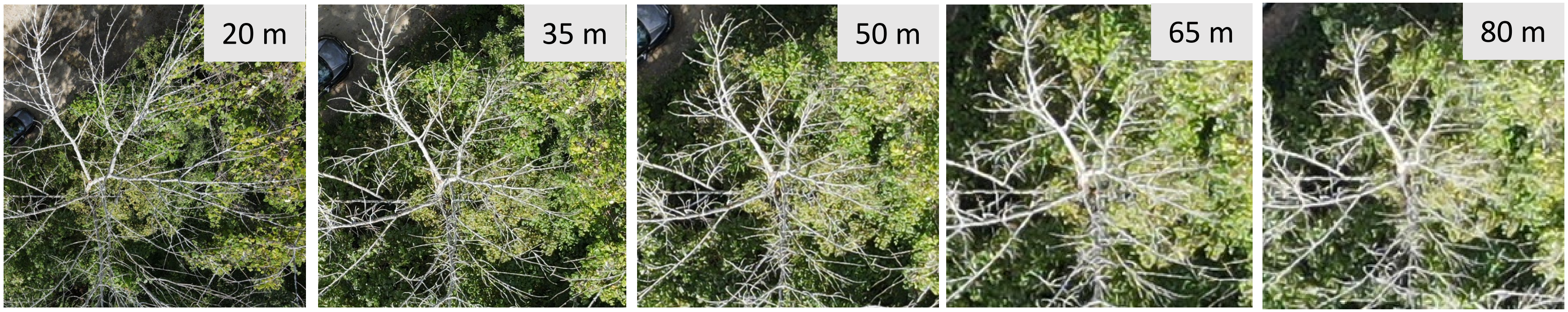

Figure S3. Resolutions of aerial images showing ash tree dieback at different drone flight altitudes. Even 80 m above the ground, aerial images show the opposite branching pattern of declining ash trees.
